# In Vivo Biomarker Imaging: Paving the Way for Precision Medicine

**DOI:** 10.3390/bios13040454

**Published:** 2023-04-03

**Authors:** Guanshu Liu, Xing Yang, Xin Zhou

**Affiliations:** 1 F.M. Kirby Research Center, Kennedy Krieger Institute, Baltimore, MD 21205, USA; 2 Russell H. Morgan Department of Radiology, Johns Hopkins University School of Medicine, Baltimore, MD 21205, USA; 3 Department of Nuclear Medicine, Peking University First Hospital, Beijing 100034, China; 4 National Center for Magnetic Resonance in Wuhan, Wuhan Institute of Physics and Mathematics, Innovative Academy for Precision Measurement Science and Technology, Chinese Academy of Sciences, Wuhan 430071, China

In vivo bioimaging has become an indispensable tool in contemporary biomedical research and medicine. Increasing evidence highlights the unparalleled role of biosensors in the specific detection of important disease biomarkers and displaying their spatial distribution in living subjects, whether invasively or non-invasively. Over the past few decades, remarkable advancements have been made in this field. While the majority of these developments have been showcased in preclinical animal models, promising clinical applications have also been reported. Ultimately, in vivo imaging and sensing will become an essential tool in precision medicine, enabling more precise diagnoses and real-time treatment monitoring by sensing underlying molecular events. This Special Issue aims to highlight recent progress in the development of in vivo imaging using clinically translatable modalities, such as magnetic resonance imaging (MRI), positron emission tomography (PET), and more. Considering the broad spectrum of technologies that exist in this field, we specifically focus on how imaging in science and chemistry can be designed based on cellular/molecular biology; this is in order to create imaging methods and probes that are able to sense previously undetectable biomarkers and translate these innovations into clinical studies to advance medicine [1].

As the theme’s title suggests, the targets for in vivo imaging are disease-specific biomarkers. To date, an abundance of biomarkers have been identified from preclinical animal models or directly from patient samples, including cell-specific biomolecules; these include receptors, glycoproteins, or metabolites, or physical and chemical changes at the cellular or tissue level caused by pathological alterations. For instance, in the study reported by Huang and colleagues, a dual-targeted peptide probe was designed for the detection of residual nasopharyngeal carcinoma and guide surgery using optical and optoacoustic imaging, targeting human epidermal growth factor receptor-2 (HER2); this is one of the members of the epidermal growth factor receptor (EGFR) family, and the scavenger receptor class B type I (SR-BI) [2]. The study demonstrated how molecular imaging approaches could be tailored to treat specific diseases for precision medicine. Pathogen-targeting capabilities can also be achieved using immune cell mimetics. Additionally, several other articles in this Special Issue showcase the in vivo imaging of changes in the extracellular pH [3], hypoxia [4], or organ function [5,6] associated with diseases.

Central to in vivo imaging and sensing is the development of sensitive and specific imaging probes, sensors, and contrast agents. In this Special Issue, advanced fluorescence imaging probes and PET/MRI agents are reported to detect cancer-related biomarkers [2,3] and parasites [7]. These imaging techniques enable the accurate delineation of disease status and treatment guidance in order to achieve elevated success rates. It is worth noting that the definition of “probes” or “sensors” is not limited to exogenous agents, and endogenous compounds can also be used. One such example is chemical exchange saturation transfer (CEST) MRI, as discussed in the review paper by Shaghaghi and Cai [8]; this has enabled the detection of pathological changes in the brain, such as ischemia and multiple sclerosis, by measuring the proton–water exchange rates of CEST reporter agents. While the landscape of endogenous agents is complex, thus making interpretation challenging, such label-free in vivo molecular imaging can be adapted into clinical settings at a much quicker pace than those approaches using synthetic imaging probes.

The advancement of powerful in vivo imaging technology necessitates not only the development of more sensitive and specific imaging agents, but also more accurate detection and analysis methods. In this Special Issue, three reports focus on technical advancements [8,9,10]. These technical advances have led to enhanced in vivo imaging platforms that aid in the observation and quantification of intricate physiological and pathological changes. Notably, the development of a holder that is suited to the functional and molecular imaging of awake mice provides more rigorous results than those obtained from anesthetized mice [9].

A wide variety of imaging modalities can be used for such molecular and functional in vivo imaging, ranging from low-tier ultrasound imaging devices (cost as low as USD 5000) to high-end positron emission tomography (PET)/magnetic resonance imaging (MRI) scanners (cost of USD > 5 million). To date, optical imaging continues to be the most extensively used imaging modality in preclinical imaging, while PET and MRI are now the methods most conventionally applied when conducting clinical studies in patients. This Special Issue incorporates both preclinical studies and clinical studies. In the meantime, multimodal imaging has become an attractive approach by which complementary imaging methods are utilized, as it provides information that a single method is unable to generate. In the Special Issue, two research papers are specifically focused on multimodal approaches [2,3], while another review paper provides a comprehensive overview of the available dual-mode imaging method, focusing on hypoxia-detection [4]. Another significant advantage of multimodal approaches is their ability to self-validate and even self-calibrate by utilizing the quantitative information detected by each of the two or more imaging methods. 

Finally, this Special Issue contains three exceptional review articles that encompass the diverse facets of in vivo imaging [4,8,11], from real-time cancer stem cell imaging [11] to quantitative analysis of human CEST MRI data [8]. Notably, the comprehensive review by Subasinghe and colleagues (spanning 72 pages (main text), 70 figures, and 400 references) provides an in-depth summary of the plethora of studies focused on hypoxia imaging [4]. This comprehensive, textbook-like review provides a brief introduction to the hallmarks of hypoxia, together with an extensive overview of the various imaging modalities utilized to detect hypoxia; this is, indeed, is invaluable for readers interested in hypoxia imaging.

In summary, the breadth of this Special Issue is vast, encompassing preclinical hardware and probe development, cell imaging, the in vivo assessment of organ function, image-guided treatment, and human imaging data analysis. This collection will prove beneficial to readers at all levels of expertise, especially those who are new to the field and eager to explore the vast possibilities of in vivo biomarker imaging.
